# Genome-wide association study for stalk lodging resistance related traits in maize (*Zea mays L.*)

**DOI:** 10.1186/s12864-023-09917-x

**Published:** 2024-01-02

**Authors:** Bangtai Wang, Meili Yang, Hua Guo, Jing Wang, Zhihong Wang, Hongwei Lu, Guiwen Qin, Jiafa Chen

**Affiliations:** 1 Hebi Academy of Agricultural Sciences, Hebi, 458031 Henan China; 2 College of Life Sciences, Henan Agricultural University, Zhengzhou, 450002 Henan China; 3 Henan Maize Breeding Engineering Technology Research Center, Hebi, 458031 Henan China

**Keywords:** Maize, Stalk traits, Lodging resistance, Dry matter content, Genome-wide association study

## Abstract

**Background:**

The stalk traits stalk diameter, stalk length, rind penetrometer resistance and dry matter content are important indicators for measuring lodging resistance.

**Results:**

In this study, 377 inbred lines were used as the basic materials, and four stalk-related traits including stalk diameter, stalk length, rind penetrometer resistance and dry matter content of the third segment of maize, were investigated at the tasseling, grain filling, and maturity stages. 461,053 high-quality SNPs which were obtained by whole genome resequencing were used for genome-wide association study. As a result of mixed linear model analysis (*P* < 9.77 × 10^–6^), 29 significant SNPs related to traits were detected, accounting for 7.19% -15.03% of phenotypic variation, among which 4, 1, 4 and 20 SNPs were found related to rind penetrometer resistance, stalk diameter, stalk length, and dry matter content respectively. Most candidate genes are related to plant element structure, signal transduction mechanisms, inorganic ion transport and metabolism, nucleotide transport and metabolism, and transporter enzyme families. Comparing mixed linear model with generalized linear model, a total of 12 candidate genes were detected repeatedly, during which the candidate gene *Zm00001d014449* were detected 5 times, with a phenotypic variation interpretation rate of 9.95% -10.84%. This gene is mainly expressed in cells with active cell division and tissue differentiation, and is involved in the formation of stalk vascular bundles and the synthesis of cell walls. Another candidate gene, *Zm00001d005300,* encodes the transcription factor MYB44, which regulates the dependence of salt stress signal phosphorylation, can effectively inhibit the accumulation of destructive reactive oxygen species, and has a certain resistance to non-biotic stress. In addition, this study also found that 10 unknown functional genes can be further Functional verification.

**Conclusions:**

This study helps to deepen the understanding of the genetic basis of traits related to maize stalk lodging resistance, and provides theoretical guidance for future maize lodging resistance breeding.

## Background

Maize (*Zea mays L.*) is one of the main food crops in the world and plays a crucial role in Chinese agricultural production [1]. In recent years, lodging has become a key factor restricting maize production, seriously affects maize yield and quality [2]. The maize lodging resistance is influenced by multiple factors, such as plant height (PH), ear height (EH), stalk diameter (SD), stalk length (SL), rind penetrometer resistance (RPR) and dry matter content (DMC), which are all significantly correlated with lodging resistance [3]. Domestic and international research on the stalk lodging resistance mostly focuses on the third internode. The stalk diameter, stalk length, epidermal puncture strength, and dry matter content can well reflect the lodging resistance of stalk and have strong correlations with lodging resistance [4].

The genetic effects of maize stalk traits are controlled by complex polygenetic quantitative traits, with both additive and dominant effects playing important roles. In the study of rind penetrometer resistance, by using genome-wide association analysis method in a natural population, scholars have detected 16 SNPs significantly related to stalk thrust resistance, and obtained 5 association candidate genes [2]; using the same method in segregating populations, two scholars found the presence of QTLs in the marker intervals SNP1950-SNP1953 and phi031-bnlg1702 on chromosome 6, respectively, and expressed them stably in multiple environments [5, 6]; some scholars have found that QTLs for rind penetrometer resistance are clustered in the chromosome segments 1, 3, and 5 of maize [7], and are associated with lignin synthesis and phenylpropanoid pathway genes [8]. However, some scholars have found that in addition to chromosome 5 or 6, QTLs for rind penetrometer resistance are distributed throughout the entire genome [9, 10], and these QTLs explain phenotypic variations ranging from 4.4% to 18.9% [11]. In terms of stalk diameter research, using isolated populations, some scholars have found that there is a main QTL in the 114.3–131.5cM region of chromosome 9 in multiple sites [7]; furthermore, through the study of the infiltration line population constructed with Ruminant Grass, 5 significant stalk-diameter associated loci were detected on chromosomes 1,2,5,6 and 7, 13 associated candidate genes were screened around the loci with the highest phenotypic contribution rate of 8.32% [12]; some scholars have also used natural populations to detect 12 SNP loci significantly associated with stalk diameter and identified 2 candidate genes [13]; 20 stalk diameter thickness consistent QTL regions were also obtained through map integration and meta-analysis methods, resulting in two candidate genes *GRMZM2G307588* and *GRMZM2G089836* for stalk diameter [14]. In terms of stalk length traits, Zhang Jianhua et al. [15] used 79 DH populations as materials to locate QTLs for stalk length on chromosome 3, bnlg1144-phi036. Li Fang et al. [12] detected significant SNP loci for stalk length on chromosomes 1 and 4.

Most of the previous studies focused on analyzing traits such as stalk puncture strength in isolated populations, but there are limited researches on stalk dry matter content in populations with wide diversity. In the present study, we used a GWAS population with wide diversity composed of 377 inbred lines for genetic analysis on stalk lodging resistance related traits. This study is aimed to explore candidate SNPs/genes controlling stalk lodging resistance at the molecular level, provide theoretical basis for genetic improvement of stalk lodging resistance traits, and further provide references for cultivating new maize varieties with strong lodging resistance.

## Results

### Phenotypic analysis of lodging resistance related traits in maize stalk

**Fig. 1:**
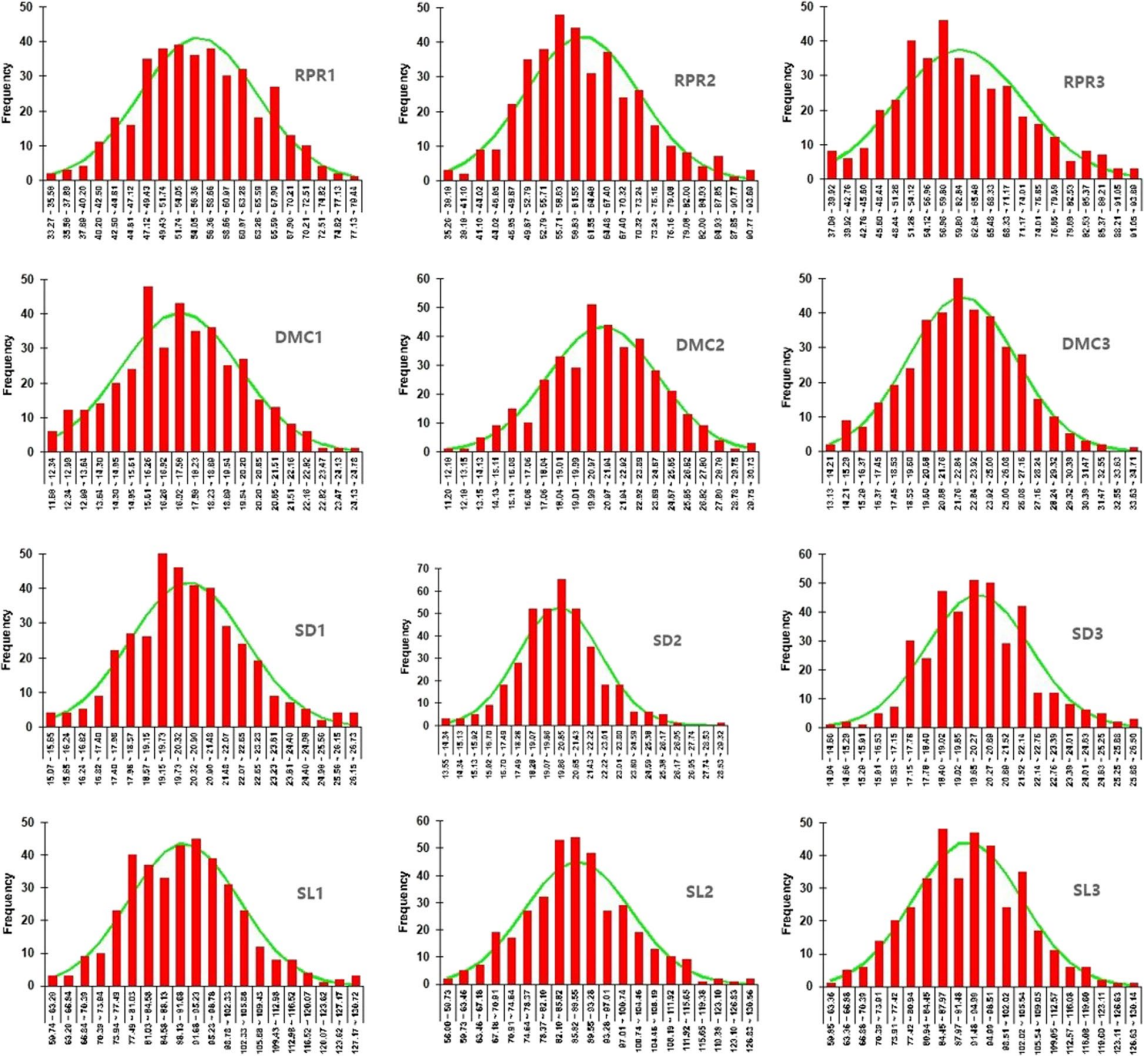
Frequency distribution map of lodging resistance related traits in maize stalk

The basic description statistics and frequency distribution of the correlation between stalk lodging resistance in different growth stages of maize are shown in Table 1 and Fig. 1. The variation range of each character is large and the phenotypic diversity is relatively rich. The absolute value of skewness and kurtosis of each character is less than 1. Combining with the histogram of character distribution frequency, it basically presents a normal distribution trend and conforms to the typical quantitative character characteristics. The generalized heritability of stalk lodging resistance related traits at different growth stages in different years is more than 60%, indicating that these traits are mainly affected by genetic factors.

**Table 1:** Statistical analysis of lodging resistance related traits in maize stalk at different growth stages

Trait	Range	Mean	CV (%)	Skewness	Kurtosis	Broad-sense heritability
RPR1(N/mm^2^)	33.27-79.44	55.77	15.1	0.07	-0.4	0.766
RPR2(N/mm^2^)	35.27-93.69	61.34	17.16	0.46	0.15	
RPR3(N/mm^2^)	37.08-93.89	61.37	18.48	0.4	-0.1	
DMC1(%)	11.68-24.78	17.27	14.11	0.06	-0.31	0.643
DMC2(%)	11.20-30.73	21.16	15.89	-0.05	-0.06	
DMC3(%)	13.13-34.71	22.57	15.99	0.02	-0.04	
SD1(mm)	15.07-26.73	20.38	10.26	0.36	0.37	0.648
SD2(mm)	13.55-29.32	20.13	11.1	0.26	0.98	
SD3(mm)	14.04-26.50	20.12	10.05	0.35	0.43	
SL1(mm)	59.74-130.72	90.78	13.46	0.34	0.21	0.714
SL2(mm)	56.00-130.56	88.21	14.05	0.32	0.36	
SL3(mm)	59.85-130.14	91.71	13.03	0.14	-0.04

### Analysis of variance and correlation among various traits of the tested inbred lines

The analysis of variance for lodging resistance related traits in 377 inbred line materials (Table 2) showed that the genotype effects of traits such as RPR, DMC, SD, SL reached a highly significant level (*P* < 0.01), indicating significant genetic variation in lodging resistance related traits in the natural population. In addition, the correlation analysis results between various traits showed (Table 3) that there was a close correlation and mutual promotion between traits such as RPR, DMC, SD, and SL in maize stalk lodging resistance. In 2016 and 2017, the correlation between each trait was basically the same. Over the course of two years, RPR reached a highly significant positive correlation with DMC, SL traits (*P* < 0.01), while RPR was positively correlated with SD, but did not reach a significant level. DMC showed a highly significant negative correlation with SD, while DMC showed a highly significant positive correlation with SL.

**Table 2:** Variance analysis of various traits in different maize inbred lines

Trait	Variation	Sum of squares	DF	SD	F
RPR	Environment	7840.1967	2	3920.0983	161.596**
	Genotype	98467.414	376	261.8814	10.795**
	Error	18242.541	752	24.2587	
DMC	Environment	5675.8017	2	2837.9008	788.143**
	Genotype	8672.4047	376	23.0649	6.406**
	Error	2707.7603	752	3.6007	
SD	Environment	16.3361	2	8.1681	5.173**
	Genotype	3872.2911	376	10.2986	6.522**
	Error	1187.448	752	1.5791	
SL	Environment	2482.4769	2	1241.2385	29.275**
	Genotype	135669.28	376	360.8225	8.51**
	Error	31884.682	752	42.3998	

*Note*: * or * * indicate a significant correlation at the 0.05 or 0.01 level.

**Table 3:** Correlation analysis of various traits in different maize inbred lines

Trait	RPR	DMC	SD	SL
RPR	1	0.55**	0.07	0.34**
DMC	0.56**	1	-0.22**	0.17**
SD	0.05	-0.36**	1	0.05
SL	0.15**	0.13**	-0.03	1

*Note*: The lower left represents the correlation between various traits in 2016, the upper right represents the correlation between various traits in 2017, and * or * * indicates a significant correlation at the 0.05 or 0.01 level.

### Genome-wide association study of stalk puncture intensity and dry matter content at different growth stages of maize

**Fig. 2:**
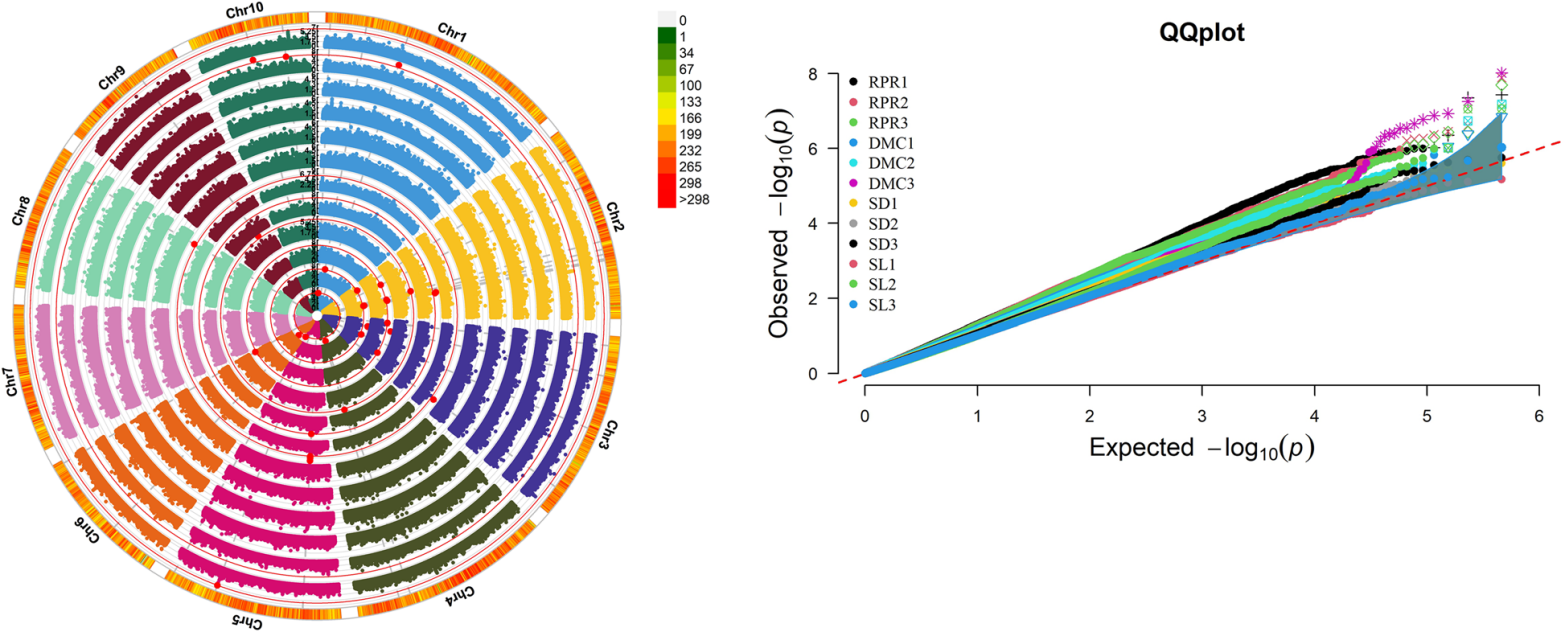
Genome-wide association study of stalk lodging resistance under GLM model Manhattan map and QQ map. From the inside to the outside, the SNP sites identified in the RPR, DMC, SD and SL of maize in different years are represented respectively; The red highlights represent the significant SNP loci of each trait at 0.01 levels, respectively; Figure b shows the QQ chart of stalk related traits of maize in different years

**Fig. 3:**
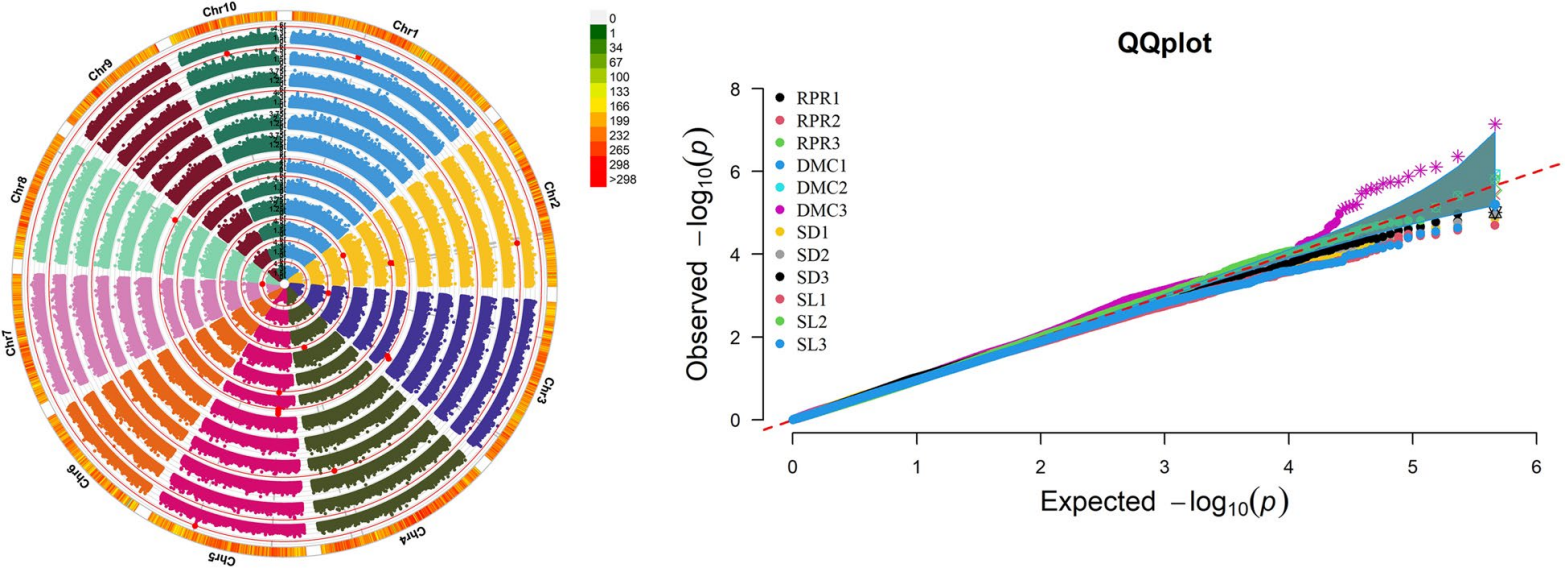
Genome-wide association study of stalk lodging resistance under MLM model Manhattan map and QQ map. Note in the figure is shown in Fig. 2

The genome-wide association study was conducted using GLM and MLM models by Tassel 5.0, and the Manhattan map and QQ map were drawn (Fig. 2 and Fig. 3). The inbred line population used in this study has rich genetic basis, which will cause a certain deviation from the confidence interval on the QQ map. SNPs exceeding the threshold are sites significantly related to the target traits of this experiment. The GLM model was used to analyze the correlation between stalks at different growth stages over two years. A total of 41 significantly associated SNPs loci were detected on 10 chromosomes of maize (Table 4). 3, 9, 7, 2, 14, 2, 0, 1, 1, and 2 SNPs were detected on chromosomes 1–10 respectively. A total of 18 SNPs (RPR1: 4, RPR2: 7, RPR3: 7) were detected related to RPR, as well as 19 SNPs (DMC1: 3, DMC2: 3, DMC3: 13) related to DMC, 4 SNPs (SL2: 3, SL3: 1) related to SL, while explaining the phenotypic variation ranged from 8.67% to 14.96%, with the highest phenotypic interpretation rate being SL2 on chromosome 10 at 70,989,765 and the lowest being RPR2 on chromosome 1 at 51,729,873. Using the MLM model to perform correlation analysis on stalk traits at different growth stages over two years, a total of 29 significantly correlated SNPs loci were detected on 10 chromosomes of maize, with 1, 4, 3, 2, 16, 0, 1, 1, 0, and 1 SNPs being detected on chromosomes 1–10 respectively. A total of 4 SNPs (RPR1: 1, RPR2: 1, RPR3: 2) were detected related to RPR, as well as 20 SNPs (DMC2: 3, DMC3: 17) related to DMC, 4 SNPs (SL2: 3, SL3: 1) related to SL, and 1 SNP (SD3: 1) related to SD, explaining a phenotypic variation range of 7.19% -15.03%, with SL2 in chromosome 10_70989765 accounting for the highest phenotypic interpretation rate, and RPR2 on chromosome 7 accounting for the lowest.

**Table 4:** Significant locus information on the correlation of maize stalk lodging resistance

Trait	Marker	Chr	Model	Pos	*p*	marker_Rsq%
RPR1	1_5925472	1	GLM	5925472	9.62E-07	10.248
RPR2	1_51729873	1	GLM	51729873	6.17E-07	7.19
SL2	1_105656256	1	GLM	105656256	8.67E-08	12.758
RPR2	2_33979628	2	GLM	33979628	5.66E-07	8.478
RPR3	2_73833386	2	GLM	73833386	8.37E-07	10.155
DMC1	2_146172998	2	GLM	146172998	8.96E-07	7.729
RPR3	2_156417807	2	GLM	156417807	5.07E-07	9.308
RPR2	2_162859353	2	GLM	162859353	5.34E-07	8.939
RPR2	2_162876327	2	GLM	162876327	2.48E-07	8.909
RPR3	2_162876327	2	GLM	162876327	3.64E-07	9.596
DMC2	2_168529088	2	GLM	168529088	6.57E-08	12.234
DMC2	2_170637158	2	GLM	170637158	1.85E-07	9.83
RPR3	3_12279836	3	GLM	12279836	7.85E-07	8.754
RPR2	3_50985869	3	GLM	50985869	1.30E-08	8.933
RPR3	3_50985869	3	GLM	50985869	2.01E-08	9.606
RPR2	3_119840946	3	GLM	119840946	4.30E-07	8.151
RPR2	3_119865757	3	GLM	119865757	3.58E-07	7.427
RPR3	3_175959350	3	GLM	175959350	9.79E-07	8.615
DMC3	3_204520247	3	GLM	204520247	5.54E-08	11.482
RPR1	4_178358908	4	GLM	178358908	4.40E-08	10.212
DMC1	4_193185052	4	GLM	193185052	4.15E-07	10.146
DMC3	5_47645572	5	GLM	47645572	2.85E-07	9.473
DMC3	5_47711262	5	GLM	47711262	2.20E-07	10.724
DMC3	5_47726081	5	GLM	47726081	3.93E-07	10.314
DMC3	5_47727153	5	GLM	47727153	1.69E-07	10.393
DMC3	5_47747099	5	GLM	47747099	9.02E-07	9.871
DMC3	5_47750439	5	GLM	47750439	6.37E-07	10.293
DMC3	5_47752006	5	GLM	47752006	1.17E-07	9.95
DMC3	5_47800595	5	GLM	47800595	9.66E-09	13.012
DMC2	5_47800595	5	GLM	47800595	9.70E-07	9.958
DMC3	5_47876150	5	GLM	47876150	2.95E-07	10.036
DMC3	5_47880130	5	GLM	47880130	4.12E-07	10.631
DMC3	5_47925170	5	GLM	47925170	4.67E-07	10.485
SL3	5_160469150	5	GLM	160469150	9.40E-07	9.172
RPR1	5_217360437	5	GLM	217360437	4.47E-07	8.571
RPR1	6_66775760	6	GLM	66775760	3.72E-08	11.587
RPR3	6_169956647	6	GLM	169956647	3.69E-07	9.647
DMC3	8_159862794	8	GLM	159862794	1.33E-07	11.605
DMC1	9_106888912	9	GLM	106888912	1.44E-07	8.732
SL2	10_70989765	10	GLM	70989765	8.78E-08	15.028
SL2	10_117358884	10	GLM	117358884	9.72E-07	10.855
SL2	1_105656256	1	MLM	105656256	1.54E-06	12.397
RPR3	2_73833386	2	MLM	73833386	7.57E-06	10.913
DMC2	2_168529088	2	MLM	168529088	7.07E-06	10.684
DMC2	2_170637158	2	MLM	170637158	1.18E-06	10.648
SL2	2_176741537	2	MLM	176741537	7.49E-06	9.657
RPR2	3_50985869	3	MLM	50985869	3.65E-06	9.437
DMC3	3_200027631	3	MLM	200027631	7.06E-06	10.199
DMC3	3_204520247	3	MLM	204520247	4.34E-07	12.685
RPR3	4_185698633	4	MLM	185698633	2.88E-06	11.224
SD3	4_201423345	4	MLM	201423345	9.69E-06	8.848
DMC3	5_47645572	5	MLM	47645572	1.76E-06	10.081
DMC3	5_47711262	5	MLM	47711262	1.81E-06	11.216
DMC3	5_47716318	5	MLM	47716318	6.60E-06	10.094
DMC3	5_47726081	5	MLM	47726081	2.61E-06	10.954
DMC3	5_47727153	5	MLM	47727153	9.38E-07	10.829
DMC3	5_47735500	5	MLM	47735500	8.41E-06	10.894
DMC3	5_47744387	5	MLM	47744387	7.54E-06	10.115
DMC3	5_47747099	5	MLM	47747099	6.11E-06	10.369
DMC3	5_47750439	5	MLM	47750439	2.95E-06	10.845
DMC3	5_47752006	5	MLM	47752006	7.82E-07	10.413
DMC3	5_47800595	5	MLM	47800595	7.17E-08	13.681
DMC2	5_47800595	5	MLM	47800595	4.85E-06	10.702
DMC3	5_47876150	5	MLM	47876150	1.31E-06	10.497
DMC3	5_47880130	5	MLM	47880130	3.47E-06	10.949
DMC3	5_47925170	5	MLM	47925170	2.56E-06	11.001
SL3	5_160469150	5	MLM	160469150	6.24E-06	9.498
RPR1	7_167499865	7	MLM	167499865	9.77E-06	8.674
DMC3	8_159862794	8	MLM	159862794	1.97E-06	11.998
SL2	10_70989765	10	MLM	70989765	3.791E-06	14.962

Further analysis revealed that 21 SNPs related to stalk lodging resistance traits were detected more than twice by independent association analystis using GLM and MLM models (Table 4), suggesting that these loci carry important genes that can stably regulate stalk related traits in different environments. Among them, four SNPs were detected more than twice by independent association analyses at different growth stages with the same stalk related traits. It is speculated that these loci may carry important genes in the growth process of corn stalk related traits, which are continuously expressed and play a role in multiple growth stages. Using GLM and MLM models, 20 SNPs related to the same stalk traits were detected more than twice by independent association analyses, indicating that candidate genes carried by these loci are more likely to be stably expressed in maize stalk related traits. These SNPs that can be significantly correlated with stalk related traits multiple times have high confidence (HC) Among them, HC-SNP site 5_47800595 was found to be significantly correlated with DMC four times during multiple growth stages in different models, and during 5_47645572-5_47925170 segments, there are 27 significant SNPs found by different models that are associated with DMC. This segment is a region where DMC related genes are enriched. Loci adjacent to 3_50985869 segment were found to be significantly correlated with RPR traits three times. The segment 2_73833386 and 2_162876327 were found to be significantly correlated with RPR twice, respectively. Loci adjacent to 1_105656256、5_160469150 and 10_70989765 were found significantly correlateed with SL. Loci adjacent to 2_168529088、2_170637158、3_204520247 and 8_159862794 were found to be significantly correlated with DMC twice. This study has not yet found the same significant SNP for different stalk traits under model analysis, that is, no one cause multiple effect significant loci were found.

### Analysis of genes associated with stalk related traits

Based on the correlation analysis between two models, candidate genes were screened and predicted within a 2.5kb range upstream and downstream of 70 significant SNPs which were associated with stalk related traits. Combined with gene functional annotation, 34 candidate genes were identified (Table 5). Among them, 22 and 12 candidate genes were obtained by the GLM model test and the MLM model test respectively, 12 candidate genes were detected more than twice. These genes may be high-confidence association genes for stalk related traits. Most of the candidate genes discovered are related to plant growth and development, carbohydrate transport and metabolism, signal transduction mechanism proteins, synthase family and cell growth regulation mechanism functions, signal transduction mechanism, inorganic ion transport and metabolism, nucleotide transport and metabolism, and transporter enzyme family. At the same time, this study also found 10 unknown functional genes, namely *Zm00001d028925*, *Zm00001d005183*, *Zm00001d040577*, *Zm00001d041441*, *Zm00001d014448*, *LOC103641209*, *Zm00001d025403*, *LOC109945001*, *Zm00001d014448* and *LOC103641209*.

**Table 5:** Prediction information of candidate gene

Trait	Marker	marker_Rsq	Chr	Pos	*p*	Phenotypic variation explanation rate%	Gene accession number	Analysis model	Gene annotation
RPR2	1_51729873	0.0719	1	51729873	6.17E-07	7.19	Zm00001d028924,Zm00001d028925	GLM	NifU-like protein 1 chloroplastic;Uncharacterized protein
RPR2	2_33979628	0.08478	2	33979628	5.66E-07	8.48	Zm00001d003157	GLM	S-adenosylmethionine decarboxylase proenzyme S-adenosylmethionine decarboxylase alpha chain S-adenosylmethionine decarboxylase beta chain
RPR2	2_162859353	0.08939	2	162859353	5.34E-07	8.94	Zm00001d005182	GLM	Charged multivesicular body protein 2a
RPR2	2_162876327	0.08909	2	162876327	2.48E-07	8.91	Zm00001d005183	GLM	Uncharacterized protein
RPR3	2_162876327	0.09596	2	162876327	3.64E-07	9.60	Zm00001d005183	GLM	Uncharacterized protein
DMC2	2_168529088	0.10684	2	168529088	6.57E-08	12.23	Zm00001d005300,Zm00001d000967,Zm00001d005301	GLM	MYB transcription factor, Transcription factor MYB44;NA;DNA binding
DMC2	2_168529088	0.12234	2	168529088	7.07E-06	10.68	Zm00001d005300, Zm00001d000967, Zm00001d005301	MLM	MYB transcription factor, Transcription factor MYB44;NA;DNA binding
DMC2	2_170637158	0.0983	2	170637158	1.85E-07	9.83	Zm00001d005351	GLM	Arogenate dehydratase, 4.2.1.91
DMC2	2_170637158	0.10648	2	170637158	1.18E-06	10.65	Zm00001d005351	MLM	Arogenate dehydratase, 4.2.1.91
RPR3	3_12279836	0.08754	3	12279836	7.85E-07	8.75	Zm00001d039695	GLM	S-adenosyl-L-methionine-dependent methyltransferase superfamily protein
RPR2	3_50985869	0.08933	3	50985869	3.65E-06	9.44	LOC109945001	MLM	Unknow
RPR2	3_50985869	0.09606	3	50985869	1.30E-08	8.93	Zm00001d040577	GLM	Uncharacterized protein
RPR3	3_50985869	0.09437	3	50985869	2.01E-08	9.61	Zm00001d040577	GLM	Uncharacterized protein
RPR2	3_119865757	0.07427	3	119865757	3.58E-07	7.43	Zm00001d041441	GLM	Unknow
RPR3	3_175959350	0.08615	3	175959350	9.79E-07	8.62	Zm00001d042665	GLM	Transcription repressor MYB6
DMC3	3_204520247	0.11482	3	204520247	5.54E-08	11.48	Zm00001d043592,Zm00001d043593	GLM	SPA1-related 3;CESA4 (CELLULOSE SYNTHASE A4)
DMC3	3_204520247	0.12685	3	204520247	4.34E-07	12.69	Zm00001d043592,Zm00001d043593	MLM	SPA1-related 3;CESA4 (CELLULOSE SYNTHASE A4)
RPR1	4_178358908	0.10212	4	178358908	4.40E-08	10.21	Zm00001d052068	GLM	Pathogenesis-related protein-1-like
SD3	4_201423345	0.08848	4	201423345	9.69E-06	8.85	Zm00001d052804	MLM	MYB transcription factor, Putative MYB DNA-binding domain superfamily protein
DMC3	5_47711262	0.10724	5	47711262	2.20E-07	10.72	Zm00001d014448	GLM	Unknow
DMC3	5_47711262	0.11216	5	47711262	1.81E-06	11.22	Zm00001d014448	MLM	Unknow
DMC3	5_47716318	0.10094	5	47716318	6.60E-06	10.09	Zm00001d014448	MLM	Unknow
DMC3	5_47744387	0.10115	5	47744387	7.54E-06	10.12	Zm00001d014449	MLM	Chromo domain-containing protein LHP1,Chromdomain-containing protein CRD101, Chromo domain-containing protein LHP1
DMC3	5_47750439	0.10293	5	47750439	6.37E-07	10.29	Zm00001d014449	GLM	Chromo domain-containing protein LHP1,Chromdomain-containing protein CRD101, Chromo domain-containing protein LHP1
DMC3	5_47750439	0.10845	5	47750439	2.95E-06	10.85	Zm00001d014449	MLM	Chromo domain-containing protein LHP1,Chromdomain-containing protein CRD101, Chromo domain-containing protein LHP1
DMC3	5_47752006	0.0995	5	47752006	1.17E-07	9.95	Zm00001d014449	GLM	Chromo domain-containing protein LHP1,Chromdomain-containing protein CRD101, Chromo domain-containing protein LHP1
DMC3	5_47752006	0.10413	5	47752006	7.82E-07	10.41	Zm00001d014449	MLM	Chromo domain-containing protein LHP1,Chromdomain-containing protein CRD101, Chromo domain-containing protein LHP1
SL3	5_160469150	0.09172	5	160469150	9.40E-07	9.17	Zm00001d014451	GLM	Glutamate receptor 3.4
SL3	5_160469150	0.09498	5	160469150	6.24E-06	9.50	Zm00001d014451	MLM	Glutamate receptor 3.4
RPR1	5_217360437	0.08571	5	217360437	4.47E-07	8.57	Zm00001d018247	GLM	Zinc finger protein
RPR3	6_169956647	0.09647	6	169956647	3.69E-07	9.65	Zm00001d039094 ;Zm00001d039095	GLM	3-ketoacyl-CoA synthase, 2.3.1.-;Uncharacterized protein
SL2	10_70989765	0.14962	10	70989765	3.79E-06	14.96	LOC103641209	MLM	Unknow
SL2	10_70989765	0.15028	10	70989765	8.78E-08	15.03	LOC103641209	GLM	Unknow
SL2	10_117358884	0.10855	10	117358884	9.72E-07	10.86	Zm00001d025403	GLM	Unknow

### Identification and functional analysis of high-confidence associated genes

Using two models, a total of 12 candidate genes were detected more than twice. Among them, *Zm00001d014449* was found to be associated with DMC five times, with a phenotypic variation interpretation rate of 9.95% -10.84%. This gene originates from the assembly of CRD101 and is associated with the LHP1 gene, which is mainly expressed in cells undergoing cell division and tissue differentiation, such as in vascular bundle synthesis, which is of great significance for maintaining cell and tissue structural specificity. *Zm00001d014449* also participates in regulating the expression of a large number of flowering genes. *Zm00001d014448* was found to be associated with DMC three times, with a phenotypic variation explanation rate of 10.09% -11.22%. The function of this gene is unknown. *LOC103641209* was found to be associated with SL twice, with phenotypic variation explanatory rates of 15.03% and 14.96%. The function of this gene is unknown. *Zm00001d005183* was found to be associated with the RPR twice, with phenotypic variation explanatory rates of 8.91% and 9.60%. The function of this gene is unknown. *Zm00001d005300*, *Zm00001d000967*, and *Zm00001d005301* were found twice to be associated with DMC, with phenotypic variation explanatory rates of 10.68% and 12.23%. *Zm00001d005300* encodes the transcription factor MYB44, Persak Helene et al. [16] found that MYB44 regulates the dependence of salt stress signal phosphorylation, can effectively inhibit the accumulation of destructive reactive oxygen species, and has a certain resistance to abiodic stress. No functional reports have been found on the *Zm00001d000967* gene. *Zm00001d005301* is related to the DNA binding domain. *Zm00001d005351* was found twice to be associated with DMC traits, with phenotypic variation explanatory rates of 9.83% and 10.65%. This gene is involved in amino acid synthesis, encodes pre phenylalanine dehydrogenase, and matches the biosynthetic pathways of phenylalanine, tyrosine, and tryptophan. *Zm00001d014451* was found twice to be associated with SL, with phenotypic variation explanatory rates of 9.17% and 9.50%. This gene belongs to the glutamic acid receptor (GLR) family gene in maize, and may be related to the opening or closing of ion channels. Under environmental stress, gene expression significantly decreases [17]. *Zm00001d040577* was found twice to be associated with the RPR, with phenotypic variation explanatory rates of 9.43% and 9.61%, and no characteristic protein was found to express by this gene. *Zm00001d043592* and *Zm00001d043593* were found to be related to DMC traits twice, with phenotypic variation interpretation rates of 11.48% and 12.69%. This gene expresses cellulose synthase A4, which is a specific signal intermediate of Phytochrome a, and is used as a light dependent inhibitor of photomorphogenesis in Arabidopsis seedlings [18].

## Discuss

### Comparative analysis of significant SNPs loci related to stalk traits

Twenty-one HC-SNPs were found by GLM and MLM tests, and then a comparative analysis was conducted according to the genetic mapping results of stalk related traits at home and abroad. Xie [4] used 301 inbred lines as experimental materials to conduct GWAS analysis on the puncture strength of corn stalks, and found a significant locus 172,176,633 on chromosome 4, which is close to the significant SNPs locus 4_178358908 related to RPR identified in this study. Liu [19] found that there were multiple QTLs at different growth stages in maize stalk puncture intensity on chromosomes 1 of 49.39Mb-58.5Mb, 2 of 156.86Mb-174.98Mb, 4 of 177.15Mb-189.04Mb, and 5 of 46.08Mb-69.55Mb and 208.1Mb-210.4Mb, respectively. The association analysis results of this study showed that RPR significant SNPs were independently detected at the 51729873 bp locus on chromosome 1, and two differentially expressed genes *Zm00001d028924* and *Zm00001d028925* were found in the candidate region of this locus. Significant SNPs for RPR and DMC traits were independently detected at 9 loci in the 156417807bp-170637158bp region of chromosome 2. Six associated gene information, including *Zm00001d005182*, *Zm00001d005183*, *Zm00001d005300*, *Zm00001d000967*, *Zm00001d005301*, and *Zm00001d0053516*, were also detected multiple times in this region. Significant RPR loci were independently detected at 178358908bp and 185698633bp on chromosome 4, and the associated gene *Zm00001d052068* was found in the candidate region of this locus. Significant DMC trait loci were independently detected at 27 loci in the 47645572bp-47925170bp region of chromosome 5, and two high reliabilities associated genes *Zm00001d014448* and *Zm00001d014449* were found in the candidate region of this locus, A significant RPR locus was independently detected at the 217360437bp locus, and an associated gene *Zm00001d018247* was found at this locus, which coincides with the interval or segment of Liu's research results. Li [20] located lignin significant SNPs near 164498311bp on chromosome 6, 172376137bp on chromosome 3, 200651833bp on chromosome 5, 152112653bp on chromosome 8, and 11457267bp on chromosome 9, respectively, and located hemicellulose significant SNPs near 11457267bp on chromosome 9_ 169624611、3_ 175959350、5_217360437 and DMC trait significance loci 8_ 159862794、9_ 106888912 is overlapping or similar. Shao [7] found that the significant SNPs loci for maize RPR and SD were clustered on chromosome 2 at 54.00Mb-75.00Mb in his study. However, in this study, significant associations with RPR were detected at both 73833386bp loci on chromosome 2 in both analysis models. Ma Qingmei et al. [2]. found significant loci for maize stalk thrust resistance on chromosome 3 at 171756718bp and chromosome 4 at 191414929bp, which were consistent with the significant loci for RPR at 3_175959350 trait and for DMC at 4_193185052 found in this study. The significant SNPs interval of stalk related traits identified in this study overlaps significantly with the correlation traits previously studied, indicating that the results of stalk related traits identified are highly reliable and suitable for further exploration of key genes related to stalk related traits. In addition, the new significant SNPs loci detected in this experiment, which are different from previous studies, especially some high-confidence significant SNPs loci and efficient significant SNPs with a phenotype interpretation rate of over 15%, are of great significance for discovering new significant loci related to stalk traits and further exploring some new associated genes.

### Analysis of genes associated with corn stalk related traits

Research has shown that there is a negative correlation between internode length and epidermal puncture strength, while there is a positive correlation between stalk diameter and stalk strength [21]. Some studies have also shown that the length of the third internode at the base of corn is significantly positively correlated with puncture intensity, while the stalk diameter is significantly negatively correlated with puncture intensity [4]. The results of this study indicate that the length of the third internode at the base of corn is significantly positively correlated with the strength of epidermal puncture over two years; the stalk diameter and epidermal puncture intensity had a positive correlation in the two-year experiment, but not significant; there is a highly significant positive correlation between dry matter content and epidermal puncture strength. Some studies have pointed out that not all corn stalk structures have a significant impact on biomass and economic coefficients, and corn basal stalk diameter has the smallest correlation coefficient with biomass, mainly due to the negative effect of stalk tip diameter reflected through basal diameter [22]. In addition, there is a significant positive correlation between stalk diameter and individual plant weight, and a negative correlation with biomass, which has not reached a significant level [23]. In this study, stalk diameter and dry matter content showed a highly significant negative correlation in the two-year data analysis. This may be due to the fact that cellulose and vascular bundles are concentrated around the epidermis in the thick material of the third section of the stalk, and most of the middle of the stalk is pithy and soft, with relatively low dry matter per unit area. The 377 inbred lines used in this study include conventional maize inbred lines, sweet glutinous maize inbred lines, and popcorn inbred lines, with a variety of types and different breeding objectives, which may have an impact on stalk correlation analysis.

Different plant tissues are composed of different types of cells. Thickened thick parietal cell form the strength of plant tissues. Genes involved in cell wall synthesis and metabolism play an important role in the formation of stalk strength. As a complex matrix, cell wall is mainly composed of polysaccharides, proteins and lignin [24, 25]. A variety of enzyme families will directly affect the metabolism and synthesis of cell wall substances. In addition, fatty acid metabolism and lipid metabolism may also affect cell wall synthesis. The structure of cell wall is also affected by the regulation and tissue of multiple transport pathways of dynamic cytoskeleton and cell wall polymer [26]. The corn stalk is mainly composed of cellulose, a small amount of sugar, inorganic salt and water. The lateral of the stalk starts to split, the cambium in the bundle grows and differentiates, and the stalk starts to thicken and extend.

The high-confidence candidate genes *Zm00001d040805*, *Zm00001d010201*, *Zm00001d021805*, *Zm00001d029050*, *Zm00001d033080*, and *Zm00001d041438* associated repetitively with SNPs markers in this study have been validated in previous studies, and are related to stalk puncture intensity and stalk correlation. They are involved in regulating metabolic substances, cell wall formation, and material transport, and can further affect stalk differentiation and cortical development. In addition, this study also found some new gene information related to stalk correlation, which is important to maintain the structure of histiocyte and participate in the regulation of root growth regulators and coenzyme families. In particular, *Zm00001d014449* is associated with LHP1 gene, which is involved in vascular bundle synthesis, and is important to maintain the specificity of cell and tissue structure; *Zm00001d010201* expresses MYB6 transcription inhibitors related to the secondary wall. Previous studies [4, 27] have confirmed that the MYB transcription factor family can be used as stalk strength related genes for in-depth research; *Zm00001d029860* participates in the metabolism of 3-hydroxyisobutyl CoA hydrolase, which affects cell wall synthesis through biological processes such as lipid transport and metabolism. Further in-depth research is needed in the next step.

## Conclusion

In this study, 461053 high-quality SNPs were used to conduct genome-wide association study on the third internode related traits of 236 maize inbred lines. With GLM and MLM, 70 significant SNPs were found to be related to stalk lodging resistance in a two-year environment, with a phenotypic variation explanation rate of 8.76% -15.03%. Among them, 21 loci were found in independent association analyses at each growth stage more than twice by different model tests, and these loci were found belonging to HC-SNP. Between 5_47645572-5_ 47925170 segments, 27 SNPs were found by different model tests to be significantly associated with DMC. The result suggests that this segment is the region where DMC related genes are enriched. Combined with gene functional annotation, a total of 34 candidate associated genes were identified among which 12 candidate genes were detected more than twice. Most of these genes are related to plant growth and development, carbohydrate transport and metabolism, signal transduction mechanisms, and synthesis transporter enzyme families. This study helps to deepen the understanding of the genetic basis of maize stalk lodging resistance related traits, and provides theoretical guidance for future maize lodging resistance breeding.

## Materials and methods

### Test materials and design

The natural test population materials composed of 377 inbred lines were selected from the domestic backbone population and the major international dominant group. A total of 400 materials of different germplasm types were collected from the International Maize and Wheat Improvement Center (CIMMYT) in Mexico, and 377 stable germplasm materials were retained through the first year of breeding. 377 materials include 231 common maize inbred lines, 58 popcorn inbred lines and 88 waxy maize inbred lines. The test materials were planted in the experimental park of the Hebi Academy of Agricultural Sciences (Hebi, Henan Province) in the summer of 2016 and 2017. The random block design is adopted in three replicates. Each plot consisted of two rows which were 5m in length, 0.6 m from the next row, with seed spacing of 0.2m. The field growth is managed according to normal agricultural production.

### Genotype and genome-wide association analysis

**Fig. 4:**
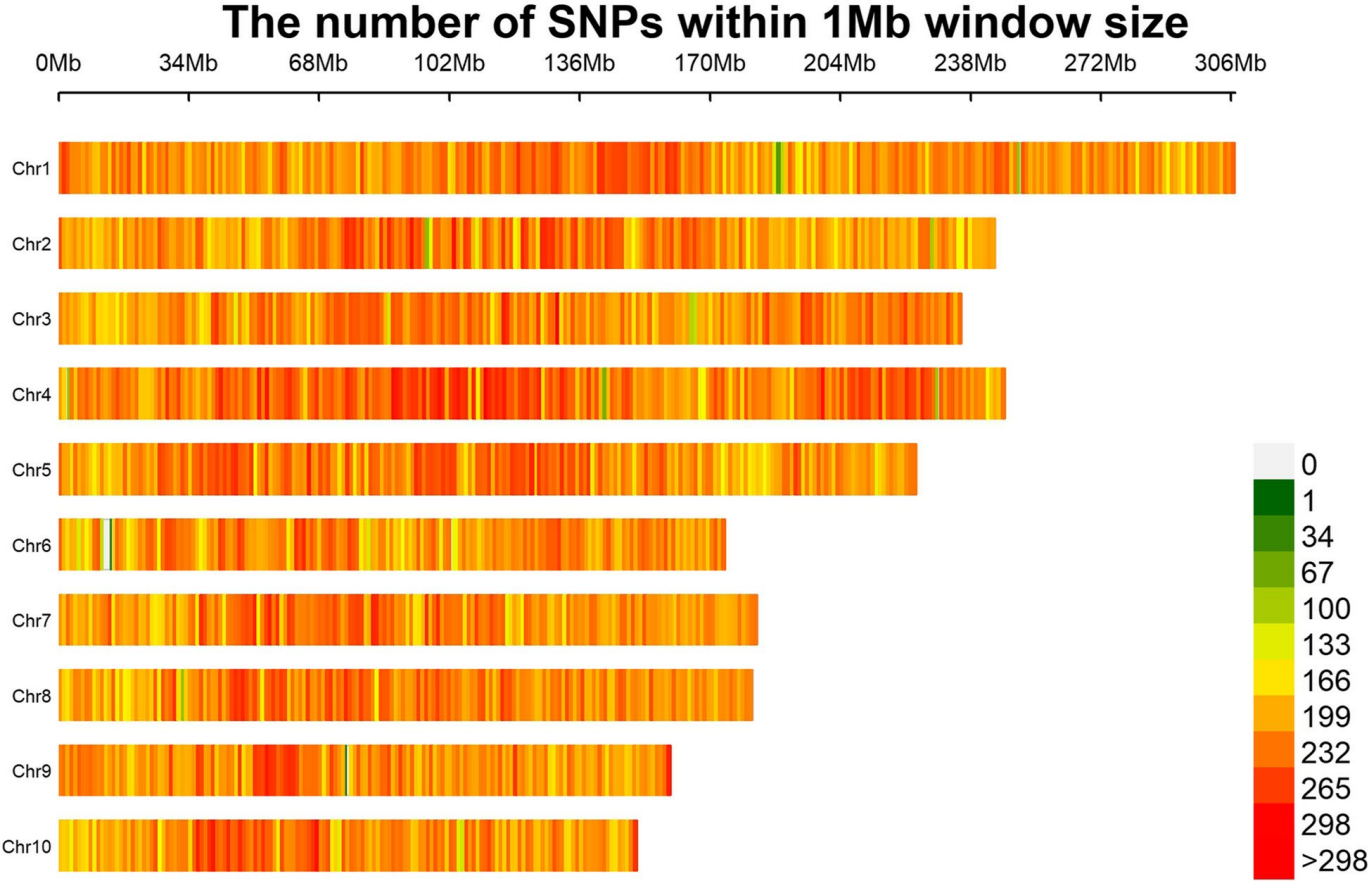
Chromosome density map containing 461,053 SNP markers

Two hundred thirty-six materials were randomly selected from the population and genomic DNA was extracted using the magnetic bead method, and then the DNA concentration, purity, and integrity were tested. Qualified DNA uses the re sequencing method to carry out genome simplified sequencing. After comparing the sequence to the B73 genome, identify the SNP, removing markers with deletion rate greater than 60% and minimum allele frequency lower than 0.05. After quality control, 461053 high-quality SNP markers were obtained for genome-wide association study (Fig. 4). At the *P* < 0.01 level, the significance of the association between SNP markers and stalk thrust resistance was determined.

Taking into account population structure and kinship, the generalized linear model (GLM) and mixed linear model (MLM) of TASSEL V5.0 software [28] were used to conduct correlation analysis on the stalk correlation at different growth stages, while the population structure-principal component analysis (PCA) was considered as a fixed effect, and kinship was considered as a random effect. According to the published Bonferroni corrected threshold GLM model with the same association group, *P* < 0.05/n was selected, while MLM model was selected with *P* < 1/n, n = number of markers [29]. Therefore, the threshold GLM model threshold for this study is *P* < 9.79 × 10^–7^, MLM model threshold is *P* < 9.77 × 10^–6^.

### Phenotypic data measurement method and statistical method

The related characters of the third above-ground stalk (stalk diameter (16SD1, 16SD2 and 16SD3 at the stage of tasseling, grain filling and maturing) were measured at the stage of tasseling, grain filling and maturing, and the number of other stalk characters in 2016 was similar to that in 2017). Select three representative plants with the same growth condition in each growth period, cut the third above-ground internode completely, and take a stalk strength appliance (Zhejiang Topp Instrument Co., Ltd., model YYD-1B) to measure the puncture resistance of corn stalk in the middle quickly, and the unit is Newton (N). Each sample shall be measured for 3 times and the mean value shall be taken. Use the digital vernier caliper to measure the stalk diameter and internode length. After measurement, the sample is blanched for 30 min at 105 °C in an air drying oven, and then dried to constant weight at 65 °C, and the dry matter content is calculated. The mean value of the three replicates represents the phenotypic value of the traits at the growth stage.

The software EXCEL2007 and DPS7.05 were used for statistical correlation analysis and heritability calculation. Calculate group PCA and Kinship using TASSEL V5.0 software. Use R "CMplot" software package to draw correlation graph (https://github.com/YinLiLin/CMplot).

### Prediction of candidate genes

According to the public database MaizeGDB (https://www.maizegdb.org/genome/), the sequence information of the B73 reference genome (B73 RefGenv4) is used as a reference to obtain the genes in the range of 2.5Kb in the upstream and downstream that are significantly associated with SNPs as the candidate genes for stalk correlation in the third internode of maize. Annotate and predict the function of candidate genes in Uniprot protein database (https://www.uniprot.org/uniprotkb/).

## Data Availability

The datasets generated during the current study are available in the [GVM000531] repository, [https://bigd.big.ac.cn/gvm/getProjectDetail?Project=GVM000531].
